# Predicting Dyslexia and Reading Speed in Adolescents from Eye Movements in Reading and Non-Reading Tasks: A Machine Learning Approach

**DOI:** 10.3390/brainsci11101337

**Published:** 2021-10-11

**Authors:** Alae Eddine El Hmimdi, Lindsey M Ward, Themis Palpanas, Zoï Kapoula

**Affiliations:** 1 Orasis Eye Analytics & Rehabilitation, CNRS Spinoff up, 12 Rue Lacretelle, 75015 Paris, France; alae-eddine.el-hmimdi@etu.u-paris.fr; 2 LIPADE, French University Institute (IUF) Laboratoire d’Informatique Paris Descartes, Université de Paris, 45 Rue Des Saints-Peres, 75006 Paris, France; themis@mi.parisdescartes.fr; 3 IRIS Lab, Neurophysiology of Binocular Motor Control and Vision, CNRS UAR 2022 Neurosciences, UFR Biomedical, University of Paris, 45 Rue des Saints Pères, 75006 Paris, France; lward@mednet.ucla.edu

**Keywords:** saccades, vergence, reading eye movements, Machine Learning, dyslexia, reading speed

## Abstract

There is evidence that abnormalities in eye movements exist during reading in dyslexic individuals. A few recent studies applied Machine Learning (ML) classifiers to such eye movement data to predict dyslexia. A general problem with these studies is that eye movement data sets are limited to reading saccades and fixations that are confounded by reading difficulty, e.g., it is unclear whether abnormalities are the consequence or the cause of reading difficulty. Recently, Ward and Kapoula used LED targets (with the REMOBI & AIDEAL method) to demonstrate abnormalities of large saccades and vergence eye movements in depth demonstrating intrinsic eye movement problems independent from reading in dyslexia. In another study, binocular eye movements were studied while reading two texts: one using the “Alouette” text, which has no meaning and requires word decoding, the other using a meaningful text. It was found the Alouette text exacerbates eye movement abnormalities in dyslexics. In this paper, we more precisely quantify the quality of such eye movement descriptors for dyslexia detection. We use the descriptors produced in the four different setups as input to multiple classifiers and compare their generalization performances. Our results demonstrate that eye movement data from the Alouette test predicts dyslexia with an accuracy of 81.25%; similarly, we were able to predict dyslexia with an accuracy of 81.25% when using data from saccades to LED targets on the Remobi device and 77.3% when using vergence movements to LED targets. Noticeably, eye movement data from the meaningful text produced the lowest accuracy (70.2%). In a subsequent analysis, ML algorithms were applied to predict reading speed based on eye movement descriptors extracted from the meaningful reading, then from Remobi saccade and vergence tests. Remobi vergence eye movement descriptors can predict reading speed even better than eye movement descriptors from the meaningful reading test.

## 1. Introduction

Reading involves complex three-dimensional binocular motor control to maintain the angle of the optic axes, or the vergence angle, stable while making saccades and fixations from left to right, allowing us to systematically capture and analyze each word. The saccades of the two eyes must be yoked so that the two eyes fixate on the same letter to obtain single fused vision. Thus, it is an oversimplification to reduce eye movement control during reading to a simple sequence of saccades and fixations. Studies focusing only on abnormalities of saccades and fixations during reading are of limited value to understand the origin of the problem. Though there have only been a few studies in the dyslexic population that have demonstrated deficits in eye movements to random targets that stimulate vergence or saccades independent from reading, such studies are essential to assess the existence of eye movement problems per se. For instance, in the dyslexic population, eye movement abnormalities in vergence have been reported using clinical subjective tests since 1988 and in binocular coordination of saccades to LED targets [1,2,3,4,5]. Most recently, Ward and Kapoula conducted a complete study of large saccades and vergence eye movements in depth using LED targets [6]. They reported specific abnormalities not only in the velocity profile of both saccade and vergence eye movements but also in certain binocular coordination aspects such as increased disconjugate post-saccadic drifts and saccade disconjugacy. The authors argue that the two phenomena are related; namely, that it is the vergence delay that compromises the quality of binocular control of saccades and fixations leading to disconjugacies. Ward and Kapoula note that such testing with three-dimensional targets in real space (in direction and in depth) is important for differential diagnosis of eye movement problems in dyslexia.

Despite evidence that demonstrates that physiologic problems with eye movements exist, dyslexia is mostly considered a learning disability rooted in phonologic processing deficits. Given these co-existing problems, it is important to establish their relationship; namely, whether eye movement deficits are the consequence or cause of poor reading and spelling problems. Despite this unclear relationship between physiologic abnormalities and phonologic processing deficits, it is clear that abnormalities in eye movements during reading depend on the text. In the dyslexic population, many studies of eye movements during reading demonstrated more frequent regressive saccades, longer fixation durations, larger or smaller saccade amplitudes [7,8,9], and poor binocular coordination of saccades and drifts [9,10]. To better characterize this relationship between text difficulty and eye movement abnormalities, Ward and Kapoula examine eye movements during two reading tasks: first, the Alouette test which requires word by word decoding of senseless text, and second, a reading of a meaningful engaging text extracted from a novel. In their study, they demonstrate that, for dyslexics, some of the aforementioned physiologic eye movement deficits during reading are exacerbated by the senseless Alouette text [11].

A few recent studies have applied ML methods to eye movement data during reading to predict dyslexia. Rello and Ballesteros [12] performed a study that examined 97 Spanish language native speakers aged 11–54 reading 12 different texts. They used a binary polynomial Support Vector Machine (SVM) classifier and achieved a classification accuracy of 80.18%. They reported that the reading time, mean fixation duration, and age have high predictive power. Benfatto et al. also employed linear SVM with sequential optimal optimization for screening dyslexia. Their study included 185 children, 97 of them with a high risk of dyslexia. Their feature set, produced using a dynamic dispersion threshold algorithm, consisted of 168 features; they categorized saccades as either progressive and regressive. A recursive feature elimination algorithm was used to identify dominant features. They achieved an accuracy of 95.6% ± 4.5% using 48 features of the original feature space [13]. Al-Edaily et al. developed Dyslexia Explorer in the Arabic language and studied 14 persons, 7 of whom were diagnosed with dyslexia. Their measurements included fixation duration, mean fixation duration in each/all Regions of Interest (ROI), total fixation count for each/all ROI, and backward saccades [14]. In contrast, Smyrnakis et al. used statistical Bayesian classifiers with various thresholds and binary correlations, focusing on a narrow age span, which is particularly critical for dyslexia diagnosis [15].

There has been one study that has compared ML analysis on eye movements during various levels of reading difficulty. Asvestopoulou et al., 2019 [16] used the same data set as Smyrnakis et al. to evaluate various ML classifiers. The linear SVM classifier on features selected by the LASSO regression at λ1SE was the classifier with the best accuracy on noise-free data. The authors concluded that feature selection, via LASSO, enabled dimensionality reduction, without compromising the accuracy. The mean and median saccade length, the number of short forward movements, and the number of multiply fixated words were the four features with the most predictive power for the baseline text, while for an easier text only the mean saccade length and the number of short forward movements were selected. The authors note that text difficulty does play an important role in the diagnosis; namely, an easy, less challenging text reduces the predictive power of the word-specific features. Though they did not compare ML analysis during reading and non-reading tasks, this study strengthens the need to apply ML to eye movements outside of reading to predict dyslexia.

Though this study presents compelling evidence for variability in predictability based on reading text difficulty, it does present some problems, including the problem of overfitting. In the second phase of learning, the testing data set that was used to evaluate the generalization ability in each step of the cross-validation was already used for feature selection, which may introduce bias. Unfortunately, these problems are inherent when dealing with small numbers of data.

There is additionally an older study with data from 185 children that were used to train an SVM to learn the same task discussed in the previous study [17]. They used Particle Swarm Optimization to compute an optimal kernel to train an SVM using cross-validation. The main drawback in this study is that the 75 descriptors extracted from the eye movement data are not presented. In addition, the eye tracker used to record eye movements had a frequency of 100 Hz which is quite low for detecting small saccades or fixation microsaccades.

Despite the many ML studies that have been conducted on dyslexic eye movements, they are limited to using data extracted only from eye movements during reading. Therefore, it is difficult to determine how text difficulty may influence ML interpretation of data or how eye movements independent of reading may be used to predict dyslexia. To summarize, there have been no studies that apply ML on a complete physiologically eye movement data set; namely, on the ensemble of eye movements tested both during complex and simple reading tasks as well as during neutral tasks such as large saccades and vergence to LED targets. Given that the predictive value of eye movements during reading has been shown to be dependent on the text, there is a real need to compare ML analysis on non-reading tests. The present study uses the data sets from two studies by Ward and Kapoula: the first testing large saccades and vergence with LED targets, and the second testing reading in two texts of varying difficulty to evaluate the capacity of eye movements to predict dyslexia [6,11]. Eye movements from all tasks were analyzed with the AIDEAL software to produce measurements of multiple physiologically valid spatiotemporal descriptors of eye movements (e.g., latency, amplitude, duration, peak, average velocity, drifts, etc.) We applied several ML models (linear and non-linear) and evaluated their differential accuracy and capacity to predict dyslexia, as well as the reading speed.

## 2. Materials and Methods

### 2.1. Participants

Given that the data used for the ML analysis is taken from published studies, please see Ward and Kapoula for full details regarding methods and materials [6,11]. In brief, eye movements were recorded during reading and non-reading tasks in 46 dyslexic adolescents (18 female, 28 male; mean age 15.52, SD 2.45) and 41 non-dyslexic adolescents (20 female, 21 male; mean age 14.78 +/− 2.44) recruited from schools in Paris. Dyslexic adolescents were diagnosed in specialized medical centers and were admitted to their schools based on their dyslexia diagnosis. Typically diagnosis by these centers include multiple testing which confers a diagnosis of visual, phonologic dyslexia, mixed dyslexia, etc. We therefore did not have children self-identify their condition. Both dyslexic and non-dyslexic adolescents had no known neurologic or psychiatric abnormalities. Typical readers had no history of reading difficulty, no visual impairment, or difficulty with near vision. Of dyslexics, 34.0% (16/47) identified that their primary issue was visual/reading based, 4.3% (2/47) auditory, 2.1% (1/47) writing, and 59.6 (28/47) were mixed or unknown. Unfortunately, our small sample size limited further analysis by category. 

Dyslexia commonly comports other comorbidities: twelve dyslexics were also diagnosed with dysorgraphia, dyscalcula, and/or dyspraxia. A total of 34 had been to an orthoptist or were currently enrolled in orthoptic rehabilitation.

The investigation adhered to the principles of the Declaration of Helsinki and was approved by our Institutional Human Experimentation Committee (CPP CNRS 18 011). Written consent was obtained from the adolescents and/or their parents after they were given an explanation about the experimental procedure. The tests were conducted by two research assistants, who were trained together using the same material and conducted the experiment together for each measurement.

### 2.2. Eye Movement Recording Device

For each adolescent, eye movements were recorded binocularly with a head-mounted video-oculography device, Pupil Core [18], enabling binocular recording at 200 Hz per eye (Pupil Labs, Berlin, Germany).

### 2.3. Ocular Motor Tests

Adolescents were asked to sit in front of a horizontal visual-acoustic REMOBI device. The device was placed at eye level so that the first arc of LEDs was 20 cm from their eyes. Each child was instructed to carefully fixate quickly and accurately on the moving LED, to not attempt to predict a pattern of motion, and to keep their head still during the 2-min test.

**Figure 1:**
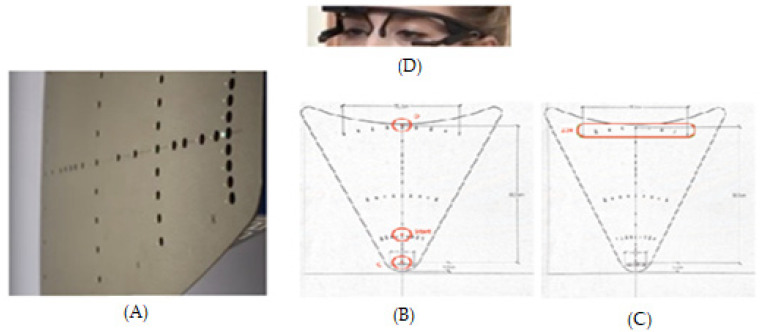
(**A**) The REMOBI device is a trapezoidal surface upon which are displayed LEDs coupled with a buzzer. The surface is placed at eye level. In (**B**) are shown in red the fixation point and the two possible target positions for the vergence test; in (**C**) is shown the central fixation target and the two possible lateral positions for the saccade test. In (**D**) the participant wears the Pupil Labs eye trackers.

Oculomotor tests were performed in mesopic light conditions. Each test was conducted in a small room in the school that was quiet and free of distractions. The red LED stimuli were displayed at different distances, laterally or in-depth, always in the horizontal plane (0°, Figure 1A). LED characteristics were: nominal frequency 626 nm, intensity 180 mCd, and diameter 3 mm. Adjacent to each LED was embedded a buzzer with the following characteristics: nominal frequency approximately 2048 Hz, sound pressure level 75 dB, diameter 12 mm.

#### 2.3.1. Vergence Test

For each trial the fixation LED (0°) lights up at 40 cm, creating a required vergence angle of 9° for a period varying from 1400 to 2000 ms (see Figure 1B). It was followed randomly by the target LED during 2000 ms, appearing always at the central axis (0°) either 20 cm (calling for a convergence movement of 8°, i.e., from 9° to 17°) or 150 cm (calling for a divergence movement of 7°, i.e., from 9° to 2°). The test contained 40 trials (20 trials of convergence, 20 of divergence, pseudo-randomly interleaved) in an overlap paradigm: after an overlap period of 200 ms following the lighting of the target LED, the fixation LED switched off.

#### 2.3.2. Saccade Test

Each trial started with the fixation central LED lighting at 40 cm from the subject for a randomized period ranging from 1400 to 2000 ms; it was followed by the lighting of the saccade target LED for 2000 ms at 20° of eccentricity, randomly chosen on the left or the right (Figure 1C). There were 40 trials (20 left, 20 right, pseudo-randomly interleaved) in an overlap paradigm.

#### 2.3.3. Reading Test

All children performed two visual reading tasks with binocular vision while seated comfortably at 40 cm viewing distance from a screen. Each adolescent was instructed to read the text out loud. First, each adolescent viewed the Alouette text, presented in 16 lines on black lines on a white background. Alouette is a text commonly used for the evaluation of reading capacity in dyslexia, as the order of the words is unusual and contains uncommon words for children. The second text was an excerpt of 15 lines in black text on white background from a children’s book (35 kilos d’espoir, Anna Gavalda, Bayard Jeunesse) targeted towards children with a reading age of 10 years.

### 2.4. Data Analysis

Data recorded with the Pupil Labs eye tracker were analyzed with AIDEAL, software developed in the IRIS laboratory. The vergence signal was derived by calculating the difference between the two eyes from the individual calibrated eye position signals (i.e., left eye–right eye). The beginning and end of the vergence movements were defined as the time point when the eye velocity exceeded or dropped below 5°/s: these criteria are standard and were applied automatically by the AIDEAL software; the program estimated the initial phasic component as the amplitude between these two initial points. It also calculated the amplitude change during the subsequent 80 ms and 160 ms. The total amplitude was calculated as the sum of the amplitude of the initial phasic component plus the 160 ms component. The total duration was calculated as the duration of the phasic component plus the subsequent 160 ms.

For saccade analysis, AIDEAL treated the conjugate signal, e.g., the L + R eye position/2. The onset and the offset of the saccade were defined as the time points where the peak velocity went above or below 10% of the peak velocity; practically, this corresponded to values above or below 40°/s (as the peak velocity of 20° saccades is typically above 40°/s). The total average velocity was defined as the ratio of total amplitude in degrees divided by time in seconds. To evaluate binocular coordination of saccades, or the disconjugacy during saccadic movements, the difference in amplitude between the left and the right eye signal was calculated. The disconjugate drift, or the difference in drift amplitude during the first 80 or 160 ms of fixation, was calculated.

Table 1 presents the results of the two reading tasks as the number of mistakes per word and the reading speed (word per minute).

### 2.5. Eye Movement Descriptors

Amplitude: the amplitude of the movement (in degrees), i.e., the change in eye position between the onset and the offset of the eye movement; the onset and offset of eye movement being determined by velocity threshold criteria as described above using AIDEAL software.

Duration of the phasic component: the time of execution of the movement (in ms), i.e., the time between the onset of the movement and its offset.

Latency: the period between the onset of the LED target and onset of the eye movement (in ms).

P-Velocity: Peak velocity of the eye movement (in degrees per sec); the peak velocity is usually achieved at the first 3rd of the trajectory of the movement.

A-velocity: Average velocity, which is obtained via the ratio of the amplitude of the movement (in degrees) over its duration (in seconds).

Drift 1: is the amplitude (in degrees) of disconjugate drift of the eyes (i.e., subtraction amplitude of the drift of the right eye from that of the left eye) for the 80 ms period starting from the offset of the movement.

Drift 2: Is the amplitude of the disconjugate drift during the 160 ms period following the offset of the movement (in degrees).

Total Amplitude: The addition of the phasic amplitude and the vergence drift during the 160 msec.

Fixation duration: the period (in ms) between two successive fixations.

Percentage of regressive saccades: “backward” saccades to the left calculated as a portion of the number of the rightward, progressive saccades.

The descriptors used for each dataset are presented in Appendix C.

### 2.6. Preprocessing of the Dataset

For each individual and each test, and each of the above-cited parameters, the AIDEAL software provides the mean value (based on 10 to 20 trials), the standard deviation, the coefficient of the variation (standard deviation/mean × 100), and the total number of trials used for each descriptor.

We considered that outlier values from AIDEAL do not occur randomly and thus convey information on the recorder gaze. For example, aberrant values could be due to blinks or artefacts of eye movement recording apparatus. We therefore chose to give these abberrances a value of 0 instead of the population average.

To remove the outliers, for the classification task (Analysis 1), we replaced the aberrant value by 0. The number of aberrant values in the 1st analysis was 0.29% for meaningful reading, 0.84% for the reading Alouette, 1.10% for the vergence, and 7.3% for the saccade dataset.

For the regression task (Analysis 2), for each dataset and each eye movement parameter, we splitthe dataset by class; then, we replaced values of the first 3% percentile, and the last 3% percentile of each eye movement parameter by its mean value. The number of outliers in the 2nd analysis was 8.7% for the vergence dataset, 9.3% for the saccade dataset, and 4.3% for the meaningful reading dataset.

Finally, we applied a random permutation to change the index of each observation in the dataset.

### 2.7. Metrics

To evaluate our ML algorithms, for the analysis 1,we used 3 metrics: accuracy, sensitivity, and specificity. In our binary classification, the sensitivity (True Positive rate) measures the proportion of positives that are correctly identified (i.e., the proportion of those who have some condition (affected) who are correctly identified as having the disease). The specificity (True Negative rate) measures the proportion of negatives that are correctly identified (i.e., the proportion of those who do not have the condition or the unaffected who are correctly identified as not having the condition).

Moreover, for the analysis 2, we used the mean absolute percentage error [19] and the Bravais–Pearson correlation metrics.

## 3. Model Fitting

### 3.1. ANALYSIS 1: Classifying Dyslexia from Eye Movement Descriptors Provided by AIDEAL

In the first ML analysis, the goal was to test the capacity of the model to predict if the person is dyslexic or not dyslexic from eye movement descriptors. Therefore, we used multiple linear classification models such as logistic regression, linear SVM, Gaussian naive Bayes, and linear discriminant analysis. However, linear models’s decisions boundaries are linear. Since, in our dataset, the samples were not fully linearly separable, to get a better decision boundary, we additionally used multiple non-linear models such as radial basis function SVM (with gamma = 0.01), k-nearest neighbors, gaussian process classifier, Quadratic discriminant analysis, and multi-layer perceptron. We also used some tree-based algorithms such as random forest classifier and decision tree classifier.

We also added Lasso regularisation whenever possible to reduce overfitting. Overfitting occurs when the models start to adjust their parameters to learn the noise present in the dataset rather than generalizing. To reduce it, one can reduce the complexity of the model by adding regularisation techniques such as the l1 norm (Lasso regularisation) and the l2 norm (Tikhonov regularisation) [20].

To summarize, our training procedure, for the four eye movements tests, we used cross-validation [21] (5 fold). At each fold, the model is fit with the train set, and its generalization ability is evaluated on the test set. We report the average test performance on the five folds.At each fold, the train set and test set were first normalized by subtracting the mean of the training set then dividing by the standard deviation of the training set. Since the size of the dataset did not allow for the creation of a representative validation set, we do not perform any hyperparameter tuning and use the default values of the different models in their scikit-learn implementation. To compare the performance of two models, we have combined the three criteria in the following way; we first use the accuracy to compare the two models. If the accuracy score is the same for two models, then the sensitivity score was used to select the best model. Finally, if the accuracy and the sensitivity score of the two models are equal, the specificity score was used. 

### 3.2. ANALYSIS 2: Predicting Reading Speed Based on Eye Movements Descriptors

In this analysis, we applied a ML algorithm on the eye movement descriptors from the three tests (saccade, vergence, and reading the meaningful text) to predict the reading speed (defined as the number of words read per minute) with a meaningful text. 

Recall that reading speed is the number of words read per minute. It is known that reading speed is slower in dyslexics, and the reading speed during the Alouette test has been found to predict dyslexia with high accuracy [22]. We used cross-validation to fit linear regression, 2-layers neural networks with 60 parameters, and support vector regression (RBF with gamma 0.01) models by applying the same procedure described in the first analysis to train the models cited above.

We have also made feature selection by keeping only the 10 most correlated features with the reading speed. The feature selection was done at each fold of the cross validation, so that we use only the training set of each iteration to select 10 features that will be used to train the model. We therefore improved the generalization ability of the models by reducing their complexity.

Once the best model was selected based on Mean Absolute Percentage Error, we retrieved its predictions on the test set during each cross-validation iteration, then applied the Bravais–Pearson correlation to test if the predicted reading speed of the child in the Alouette test based on eye movement descriptors was positively correlated with measured reading speed.

## 4. Result

### 4.1. Analysis 1

For the four oculomotor tests, the best linear classifier (in terms of accuracy) was the Logistic regression. The best non-linear classifier was the SVM with an RBF kernel. 

In Table 2 and Table 3 we present the score of each best model so that we can compare the ability of the descriptors extracted from each oculomotor test to predict dyslexia using the family of models that we have tested. Moreover, the accuracy of all the other trained models are presented in the Appendix D.

The eye movement parameters extracted by AIDEAL can predict dyslexia by using the SVM and logistic regression classifiers with an accuracy of 71.57% and 70.2% when it was trained on the data from the meaningful reading test, 68.42% and 77.3% when it was trained on the REMOBI vergence dataset, 80.0% and 81.25% when it was trained on the REMOBI saccade dataset, and 80.0% and 81.25% when it was trained on the Alouette reading dataset.

The best SVM model in terms of sensitivity is the model trained on the data from the Alouette reading test with a score of 85.0%. The best logistic regression model in terms of sensitivity is also the model trained on the REMOBI saccade test with a score of 86.94%, demonstrating that the Alouette test could also be used to detect dyslexia.

The best model in terms of specificity is the model trained on saccades produced by the Remobi table with a specificity of 82.5%, which means that the Remobi saccade test could be used to detect the non-dyslexic population. Note that the performances we report are obtained without hyper parameter tuning. A larger dataset would likely allow even better performances by tuning the hyper parameters on a separate validation set. 

To investigate the signification of the reported result, we implemented a label permutation test. For each dataset we have fitted the two selected models after having applied a random permutation to the labels of the entire dataset. We have repeated this procedure 1000 times.

Then, we have computed the probability of getting an accuracy at least equal to the scores presented in the Table 2 and Table 3 in order to estimate the significance of the reported result.

**Figure 2:**
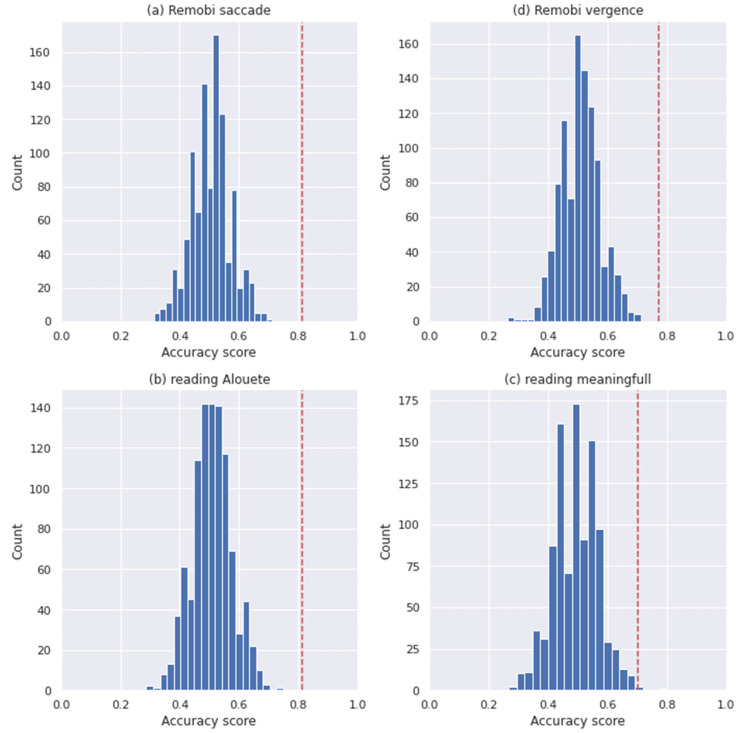
Logistic regression test permutation histograms for Analysis 1, the red line indicated the reported score. (**a**) the histogram of the Remobi saccade dataset. (**b**) the histogram of the Remobi vergence dataset. (**c**) the histogram of the reading Alouette, (**d**) the histogram of the reading meaningful.

**Figure 3:**
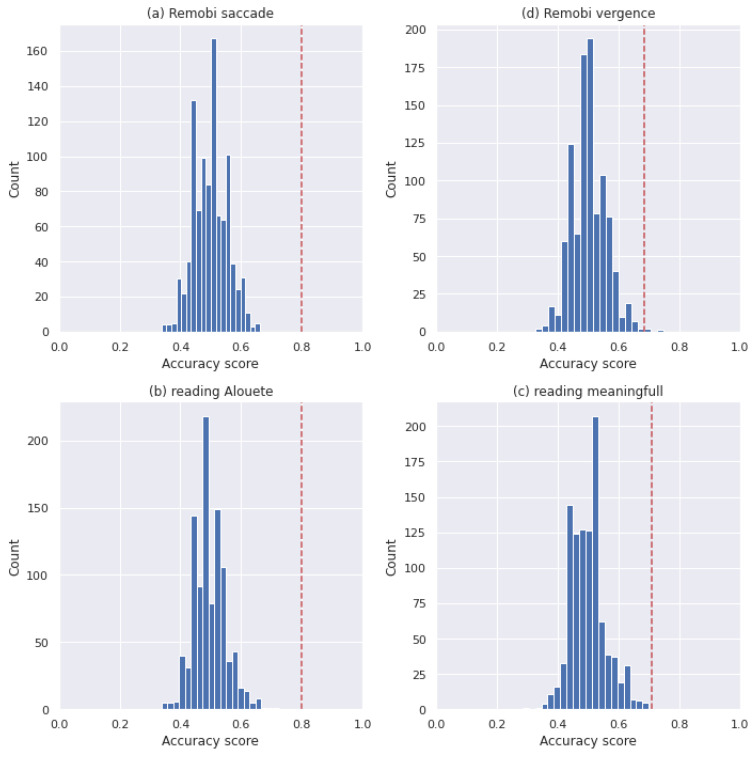
SVM test permutation histograms for Analysis 1; the red line indicates the reported score. (**a**) The histogram of the Remobi saccade dataset, (**b**) the histogram of the Remobi vergence dataset, (**c**) the histogram of the reading Alouette, (**d**) the histogram of the reading meaningful.

The significance of the reported results are presented in Table 4 and the histogram of the scores of each model for each dataset are presented in Figure 2 and Figure 3.

### 4.2. Analysis 2

The best model for predicting the meaningful reading speed from the eye movement parameters of the Remobi saccade test was the RBF SVM. The model achieved a mean absolute percentage error of 26.50%, and the predicted speed was correlated with the real speed of 0.46.

For the second task, which was predicting the meaningful reading speed from the eye movement parameters of the REMOBI Vergence test, the best model was the same as of the previous task. This model achieved a mean absolute percentage error of 16.78%, and the predicted speed was correlated with the real speed with a correlation coefficient of 0.56.

Finally, for the last task, which was predicting the meaningful reading speed from the eye movement parameters of the meaningful reading test itself, the best model was the same as the previous tasks. The model achieved a mean absolute percentage error of 22.2%, and the predicted speed was correlated with the real speed with a correlation coefficient of 0.56.

**Figure 4:**
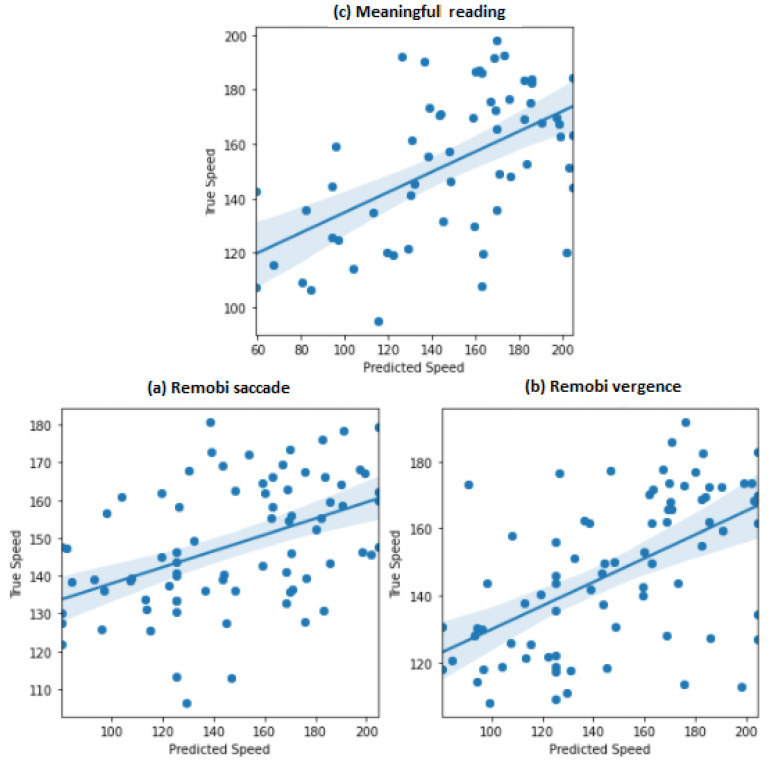
Correlation between true speed vs. predicted speed of the three tasks for the analysis 2: (**a**) Remobi saccade. (**b**) Remobi vergence. (**c**) Meaningful lecture.

In Figure 4, we present the correlation between true speed and the predicted speed of the three tasks. The accuracy and the mean percentage error for each of the four tasks are presented in Table 5:

For each task, the eye movement parameters selected for at least 4 folds are presented in Table 6 and Table 7, the correlation is computed on the base of the entire dataset.

## 5. Discussion

ML has been applied to numerous fields including health and neuroscience. Research on the neurology of eye movements has been a pioneering field of cognitive neuroscience for almost a century. Nevertheless, the application of Machine or Deep Learning (DL) in the field of dyslexia and oculomotor movements is rather scarce. This could be related to the fact that eye movement researchers have already extensively characterized many spatio-temporal parameters of eye movements in relation to their specific functions and their neural substrates. Indeed, eye movement control is the best-understood sensorimotor system in neurosciences.

Yet, ML/DL can bring some new insights and advantages: first, such an approach is more data-driven. Second, such methods apply a variety of different models including linear and non-linear. Therefore, our approach was to apply ML on data to quantify more precisely the quality of such eye movement descriptors for dyslexia detection. To this end, we used the descriptors produced from four eye movement tests as input to common ML classifiers to compare their generalization performances.

A review of the existing literature in this field reveals that the few existing prior ML studies use a limited set of data applied to reading concerning only saccades and fixations [23]. Therefore, the present study aims to use ML to further the fields of neuroscience, dyslexia, and ML itself. First, in its very practice, our study unites the fields of computer science and neuroscience. Second, our study uses a complete data set carefully assembled from cohorts diagnosed with dyslexia by medical reference centers in France. Third, the data used covers almost the entire ensemble of physiologic eye movements we make to explore the three-dimensional space, namely, saccades and vergence eye movements to LED targets using the REMOBI device, and reading of two types of text: a meaningful text and the Alouette text used by orthophonists and psychologists in France to diagnose dyslexia. Another advantage of the study is that we have used many biologic descriptors of the eye movements (latency, peak, average velocity, amplitude, duration, coordination, drifts, disconjugacy) that are well established in the field of the neurology of eye movements. Such descriptors enable us to describe not only the eye displacement onset and offset of the saccade and fixation but also the trajectory of the movements themselves. The ML models, pre-processing, and post-evaluation methods were carefully done to provide plausible physiological results, uniting neuro-physiologic and ML expertise.

The results show that an ML approach on such data is useful and provides convincing power to determine dyslexic vs. non-dyslexic classifications. Further, the fact that we were able to create an ML approach that could classify dyslexic from non-dyslexic adolescents based on eye movements during reading is a novel result, shedding light on the importance of eye movements during reading, which is often overlooked by many language-oriented researchers who consider reading solely to be a language issue. It is remarkable that the power of classification is modulated according to text (senseless or meaningful); that the meaningless text stresses dyslexics’ more fragile oculomotor system reveals how we can classify dyslexic’s eye movements based on their deficits. Perhaps the most remarkable finding is that we were able to powerfully classify large saccades and vergence eye movements to LED targets into dyslexic and non-dyslexic categories. Finally, we were also able to predict reading speed based on eye movement descriptors from tests with LED targets, an approach that has not been previously seen. Our results based on ML classification confirm previous studies that have performed classic group comparison analysis [6,11]. The present study confirms classification power with multiple linear and non-linear models with significant results. Clinically, this study also raises the possibility of a ML diagnostic help assistant, the development of which under progress.

Finally, we should address the physiologic significance of such results. Recall that eye movements are essential for reading during the learning process and exploration of the environment. There is ample evidence in the neuroscience field that eye movements are related to attention and cognition, most frequently referenced via the well-established theory of the motor basis of attention introduced by Rizolatti [24]. Prior research from our team has expanded this theory to a broader motor basis that includes not only saccades as was introduced by Rizolatti but also vergence eye movements and accommodation [25,26,27]. As previously mentioned, eye movement control during reading is complex, binocular, and three-dimensional, involving the control of movements of the two eyes in multiple directions (horizontal and vertical) and in depth (vergence). Though the motor theory of visual attention is generally accepted in neurosciences, when it comes to reading, such physiologic approaches are rarely considered in favor of the predominantly phonologic and language-based approach.

Typical arguments against such a physiologic approach are that people with visual problems (for example, the elderly) can oftentimes still read well. Although this is often the case, senior readers who develop problems later in life can usually compensate for visual and motor control issues. People such as dyslexic adolescents, who experience these deficits from an early age during development, before coordination of oculomotor movements is truly learned and cemented, are unable to compensate for these deficits, which can have devastating consequences for reading throughout their lives.

## 6. Limitations 

As mentioned in our methods section, 34% of the dyslexic population studied had visual/reading predominant dyslexia, 4.3% were classified as auditory dyslexics, and 59.6% were mixed or unknown. We were unfortunately unable to further break down our analysis to study each type of dyslexia given our relatively small sample size. In addition, the concept of breaking dyslexia down further into different subtypes is controversial: a number of articles detail different methodologies to determine dyslexic subgroups, which are not routinely described in necessarily the same fashion (see [28,29,30] for a small sample of such articles). Further, one such paper describes how dyslexics most often have a mixed picture with multiple phenotypes [31]. Finally, it is open to question whether it is even possible to accurately categorize further subgroups of dyslexia [32].

In any case, despite the fact that the dyslexic population was largely classified as visual predominant and mixed/unknown, it was still possible for us to determine that the larger dyslexic group demonstrated significantly different oculomotor movements as compared to the control group, giving the impression that oculomotor abnormalities are important distinguishing factor in the dyslexic population as a whole. 

Further interdisciplinary research with larger population and use of different types of reading tests should aim to establish specific link of the eye movement disorders with morphological, phonological, syntactic, or semantic errors.

## 7. Controversy about Dyslexia 

In his article “Does dyslexia exist” [33], Stein argues that is important to test auditory and temporal processing in the potential dyslexic child in addition to the standard phonological testing in order to better distinguish true dyslexia from pathologies that may cause other types of phonological deficits. 

In his article “What is developmental dyslexia?” [34], Stein reiterates that a purely phonologic etiology of dyslexia excludes involvement of visual processing or other reasoning skills. Alternative to a purely phonologic theory, he proposes the magnocellular deficit theory, which could explain deficits in both visual and auditory sequential processing in dyslexics. 

A more provocative view by Elliott and Gibbs [35] criticizes the differentiation between dyslexics, poor readers, and normal readers, writing that there is no solid scientific basis for such differentiation and appropriate treatment can even be hindered by such a dichotomy. 

Returning to the present study, all dyslexic participants were found to have difficulties with literacy. Further, problems of binocular motor control were systematically present, suggesting that dyslexia does indeed have some component of visual deficiency. Indeed, until 1950, dyslexia was primarily categorized as a visual disability. Unfortunately, this view is also quite reductive in its singularity. 

A more comprehensive physiologic approach may be that poor sensorimotor binocular control prevents a clear, unified vision of letters and words. Reciprocally, these motor deficits may persist due to impaired visual input, given vision and eye movements are intimately linked. Such an explanation does not exclude other factors such as deficiencies in auditory and sequential temporal processing, such as those mentioned by Stein. If attention is a byproduct of eye movements, particularly of movements in depth, this theory could be expanded to other multisensory processes, including audiovisual integration. Essentially, there is a need for a multidisciplinary approach in which we can build efficient multisensory retraining programs to improve eye movements and attention.

## 8. Conclusions

Our results demonstrate that eye movement parameters from the saccade and vergence Remobi tests can successfully predict both dyslexia diagnosis and reading speed; further studies with larger populations are of interest. The ML results applied to reading itself are highly dependent on the type of text read. The results from ML presented here are in full agreement with our prior studies, showing statistically significant differences in many eye movement parameters with the classic group. The ML approach provides quantification and prediction power, opening the possibility of creating an early diagnostic tool to detect dyslexia independent of reading.

## 9. Patents

Zoi Kapoula has applied for patents for the technology used to conduct this experiment: REMOBI table (patent US8851669, WO2011073288); AIDEAL analysis software (EP20306166.8, 7 October 2020; EP20306164.3, 7 October 2020—Europe).

## Figures and Tables

**Table 1:** Dyslexic vs. Non-Dyslexic performance for each reading task.

	Dyslexic		Non-Dyslexic	
	Median	SD	Median	SD
Mistakes per word (35 Kilos d’Espoir)	0.03	0.03	0.015	0.01
Words per Minute (35 Kilos d’Espoir)	124	33	176	34
Mistakes per word (L’Alouette)	0.06	0.06	0.026	0.02
Words per Minute (L’Alouette)	92	109	136	29

**Table 2:** Support Vector Machine scores for Analysis 1.

Metric	Reading Alouette	Reading Meaningful	Remobi Saccade	Remobi Vergence
accuracy	80.0%	71.57%	80.0%	68.42%
sensitivity	82.5%	54.29%	82.5%	67.5%
specificity	77.5%	76.67%	77.5%	69.17%

**Table 3:** Logistic regression scores for Analysis 1.

Metric	Reading Alouette	Reading Meaningful	Remobi Saccade	Remobi Vergence
accuracy	81.25%	70.2%	81.25%	77.3%
sensitivity	85.0%	62.38%	80.0%	80.0%
specificity	77.5%	76.67%	82.5%	74.17%

**Table 4:** Significance of reported results for Analysis 1.

Model	Dataset	*p*-Value
	Remobi saccade	0.000
	Remobi vergence	0.000
Logistic	Reading alouette	0.000
regression	Reading meaningfull	0.002
	Remobi saccade	0.000
	Remobi vergence	0.003
SVM	Reading alouette	0.000
RBF	Reading meaningfull	0.000

**Table 5:** Mean percentage error and correlation metrics for the analysis 2.

Metric	Remobi Saccade	Remobi Vergence	Reading Meaningful
correlation	0.46	0.56	0.56
mpe	20.45%	16.78%	22.2%

**Table 6:** The eye movement parameters selected for the Remobi saccade and vergence datasets.

Model	Feature Parameters	Correlation	*p*-Value
Remobi Saccade test	Duration left	−0.20	0.055
	Duration left std	−0.28	0.0062
	Pvelocity left	−0.23	0.02616
	Pvelocity left std	−0.21	0.0449
	Avelocity left	0.25	0.0137
	Avelocity left std	−0.26	0.0112
	Duration right	−0.23	0.0248
	Duration std right	−0.33	0.0013
	Pvelocity right	−0.21	0.0445
Remobi Vergence test	Divergence Duration	−0.26	0.0115
	Divergence Avelocity	0.33	0.0012
	Convergence Avelocity	0.32	0.002
	Convergence AVelocity CoV	−0.31	0.0026
	Convergence Drift 1	0.32	0.0018
	Convergence Drift1 CoV	−0.28	0.0069
	Convergence Drift 2	0.33	0.0014
	Convergence Drift2 CoV	−0.25	0.0137

**Table 7:** The eye movement parameters selected for the Alouette and meaningful lecture data sets.

Model	Feature Parameters	Correlation	*p*-Value
Meaningful lecture test	Reading Left PVelovity CoV	0.53	0.000008
	Reading Left Avelocity	0.53	0.00000008
	Reading Left AVelocity CoV	−0.50	0.0000005
	Reading Left Drift CoV	−0.40	0.00007
	Reading Right Amplitude	0.47	0.000004
	Reading Right Avelocity	0.52	0.0000002
	Reading Total Number Left Sacc	−0.47	0.000003
	Reading Total Number Right Sacc	−0.54	0.0000000005

**Figure A1:**
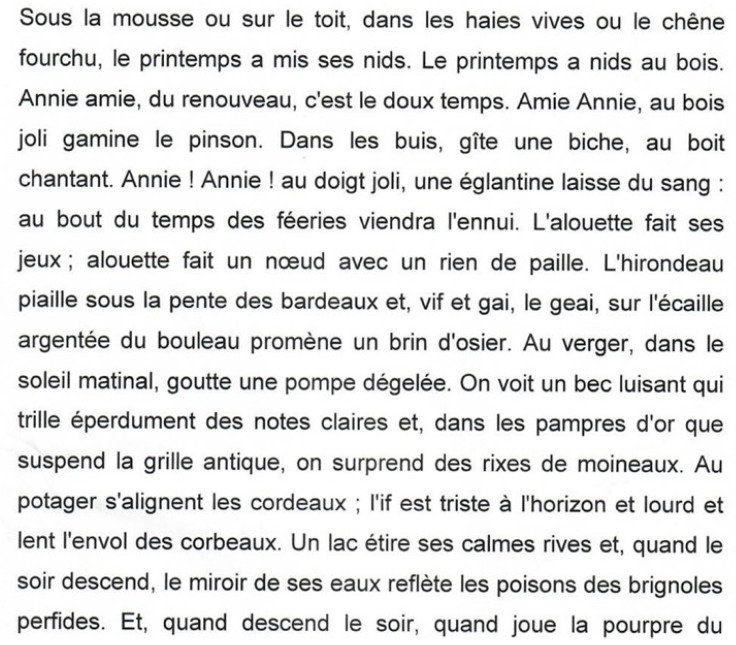
L’Alouette Text.

**Figure A2:**
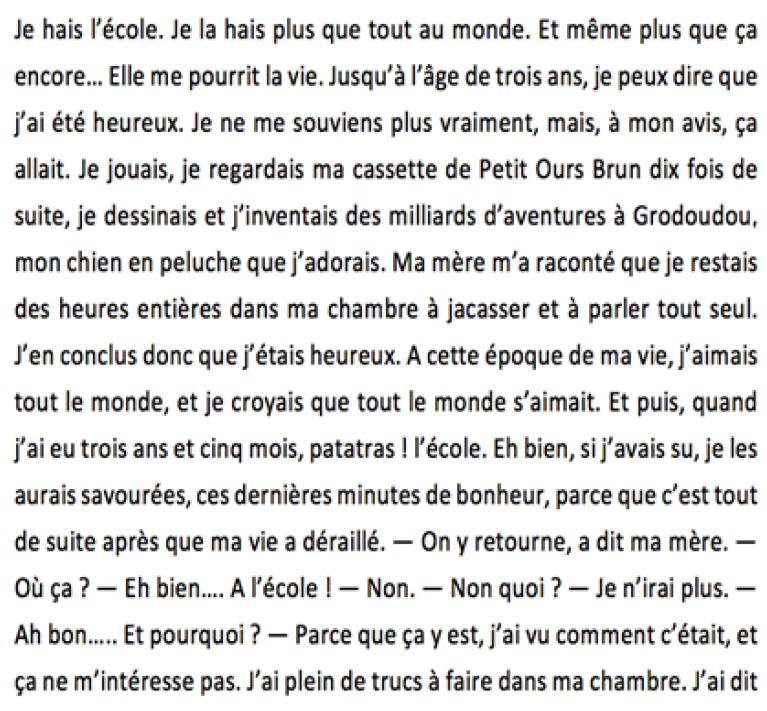
35. Kilos d’Espoir Text.

**Figure A3:**
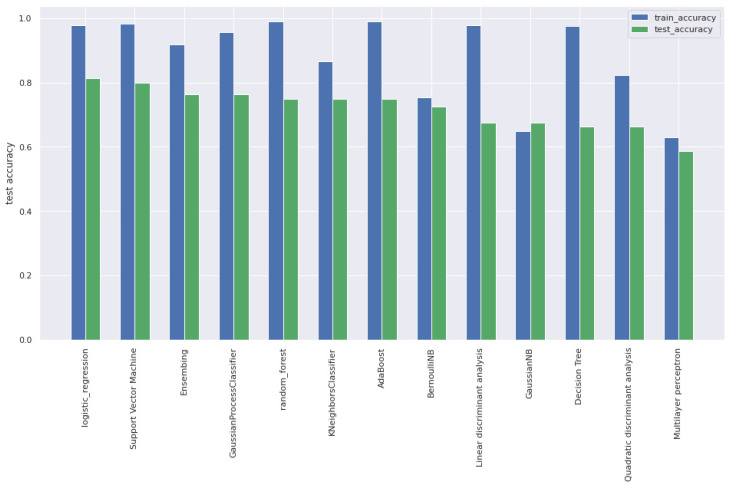
Pair plot of all the score of the analysis 1—Remobi saccade dataset.

**Figure A4:**
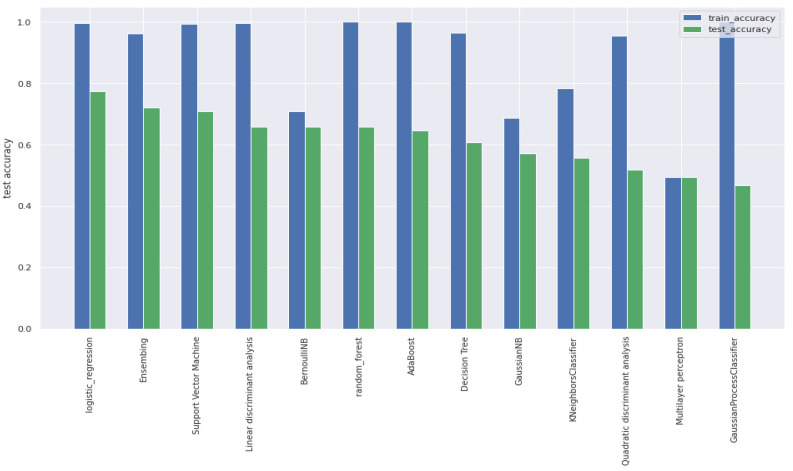
Pair plot of all the score of the analysis 1—Remobi vergence dataset.

**Figure A5:**
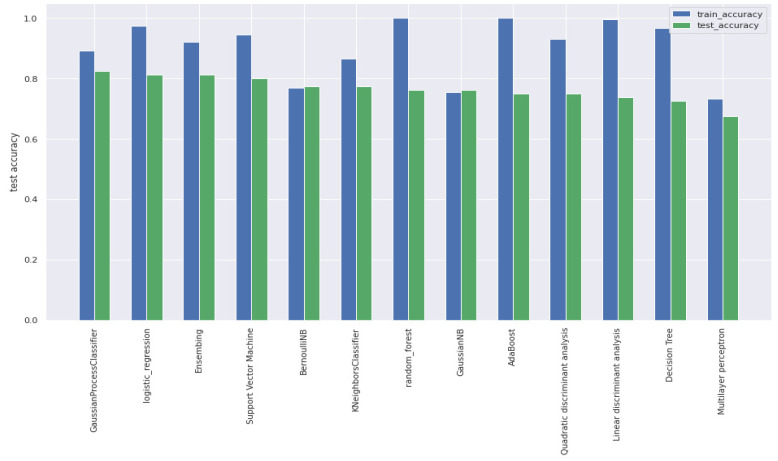
Pair plot of all the score of the analysis 1—Remobi Alouette reading dataset.

**Figure A6:**
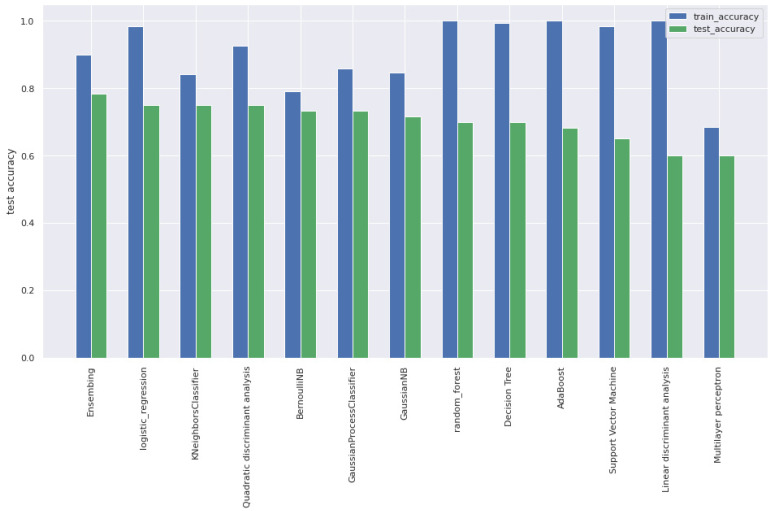
Pair plot of all the score of the analysis 1—Remobi Meaningful reading dataset.

## Data Availability

The datasets generated during and/or analyzed during the current study are available from the corresponding author on reasonable request.

## Appendix C

**Table A1:** List of the descriptor used for each model.

Dataset.	Eye Movement Descriptors
Remobi Saccade	Amplitude, Duration of the phasic component, Latency, P-Velocity, A-velocity, Drift 1, Drift 2
Remobi Vergence	Amplitude, Duration of the phasic component, Latency, P-Velocity, Drift 1, Drift 2, Total Amplitude, Average velocity
Meaningful Reading	Amplitude, Duration of movement, Latency, P-Velocity, A-velocity, Drift 1, Drift 2, Fixation duration, Percentage of regressive saccades
Alouette Reading	Amplitude, Duration of movement, Latency, P-Velocity, A-velocity, Drift 1, Drift 2, Fixation duration, Percentage of regressive saccades
